# Weight-loss behaviors before pregnancy associate with increased risk of postpartum depression from the Japan Environment and Children’s Study

**DOI:** 10.1038/s41598-023-34547-4

**Published:** 2023-05-05

**Authors:** Saki Taniguchi, Toshio Masumoto, Youichi Kurozawa

**Affiliations:** 1 Tottori Regional Center of The Japan Environment and Children’s Study, Faculty of Medicine, Tottori University, Tottori, Japan; 2 Division of Health Administration and Promotion, Department of Social Medicine, Faculty of Medicine, Tottori University, Tottori, Japan

**Keywords:** Epidemiology, Weight management, Risk factors

## Abstract

No studies showed the association between weight-loss behaviors before pregnancy and postpartum depression (PPD). We analyzed data from the nation-wide birth cohort study, the Japan Environment and Children’s Study. Self-administrated questionnaires answered by 62,446 women was analyzed using logistic regression analysis. PPD was assessed using the Edinburgh Postnatal Depression Scale at 1 month after delivery. Women using at least one weight-loss method had higher risk of PPD than women using no weight-loss methods [women without antenatal psychological distress according to Kessler 6-Item Psychological Distress score: adjusted odds ratio (aOR) 1.318, 95% confidence interval (CI) 1.246–1.394; women with antenatal psychological distress: aOR 1.250, 95% CI 0.999–1.565]. Using extremely unhealthy weight-loss methods was associated with PPD compared with no use of each weight-loss method (vomiting after eating: aOR 1.743, 95% CI 1.465–2.065; smoking: aOR 1.432, 95% CI 1.287–1.591; taking diet pills: aOR 1.308, 95% CI 1.122–1.520). The association between weight-loss behaviors and PPD varied according to pre-pregnancy BMI. In normal-weight women, the weight-loss method score, which indicates the degree of weight-loss method use, was associated with PPD. These results indicate using weight-loss methods before pregnancy is associated with an increased risk of PPD in Japanese women.

## Introduction

Extreme weight-loss behavior among young women is a serious problem in Japan. The National Health and Nutrition Survey^1^ reported that the rate of underweight status [body mass index (BMI) < 18.5 kg/m^2^] among women of childbearing age in their 20 s is 20.7% and the rate of normal weight (BMI 18.5–24.9 kg/m^2^) is 70.4%. Among women in their 30 s, 16.4% are underweight and 68.7% are normal weight. These results indicate that most women have no need to lose weight. However, because of the influence of media^2,3^, fashion^4^, and body image distortion, unbalanced eating habits and extreme weight-loss behaviors are common, even among women with no need to lose weight. Women who attempt to lose weight in an unhealthy manner have an increased risk of mental and physical health problems and unfavorable eating habits^4–6^. Moreover, it is reported that insufficient gestational weight gain is a risk factor for symptoms of postpartum depression (PPD) among women with a pre-pregnancy BMI of 20.0 to < 23.0 kg/m^2^^7^. Therefore, it is important to clarify whether the weight-loss method used by women, including pregnant women, is associated with mental and physical health problems.

As reported in a World Mental Health Japan survey^8^, risk factors for depression include domestic violence in childhood, mild mental illness, and physical illness. Several recent studies have examined the relationships between depressive symptoms and unhealthy weight-loss behaviors including smoking, vomiting, using laxatives, skipping meals, and taking diet pills. For example, women with inaccurate body perception tend to engage in unhealthy weight-loss behaviors, which can lead to depression^9^. Furthermore, a study using data from the National Health and Nutrition Examination conducted from 2005 to 2014 reported that women using at least one unhealthy weight-loss method had a tendency to be depressed^10^. Thus, it is important to provide correct information about unhealthy weight-loss behaviors to help prevent mental problems among women.

Some women experience depressive symptoms after pregnancy, and some women develop PPD. Recently, the estimated prevalence rate of PPD is 15.1% among pregnant Japanese women at 1 month after delivery^11^ and 17.7% worldwide^12^. PPD begins within 4 weeks after delivery, with symptoms including sadness, anxiety, irritability, loss of interest, anorexia, appetite disturbance, excessive or lack of concern for the baby, and constant fatigue^13^. These symptoms adversely affect mother–infant interaction, breastfeeding, sleep routines, infant health care, and safety practices^14^. Additionally, children who have mothers with PPD exhibit an increased risk of behavioral problems in childhood, depression, and a decline in mathematics performance during adolescence^15^. Therefore, it is important to detect and treat PPD among postpartum women in the early stages.

To prevent PPD using a public health approach, risk factors of PPD should be investigated in detail. According to meta-analyses and systematic reviews^16–19^, risk factors of PPD include marital status, poor conjugal relations, a history of depression, psychological disturbance during pregnancy, stressful life events, unplanned pregnancy, low levels of social support, socioeconomic status, low self-esteem, neuroticism, and obstetric factors. Added to these factors, smoking^20^ and intimate partner violence^21^ have also been reported as risk factors. Although the risk factors of PPD have been well investigated, no studies have examined the association between weight-loss behaviors during the year before the current pregnancy and PPD. Considering the association between depression and unhealthy weight-loss behavior, we hypothesized that unhealthy weight-loss behaviors are risk factors of PPD. Furthermore, because most women tend to try multiple weight-loss methods to lose weight, we hypothesized that women who engaged in multiple weight-loss methods may have a higher risk of PPD. To investigate these hypotheses, we analyzed data from the Japan Environment and Children’s Study (JECS), a nationwide birth cohort study in Japan.

## Methods

### Study design and participants

The aim of the JECS, an ongoing prospective birth cohort study that began in 2011, is to evaluate the effects of various environmental factors on the health and development of children. Kawamoto et al. (2014) reported the JECS protocol and its details^22^. In brief, the JECS recruited 97,413 pregnant women living in the study areas (15 regional centers, 19 prefectures) between January 2011 and March 2014. The JECS protocol was reviewed and approved by the Ministry of the Environment's Institutional Review Board on Epidemiological Studies and the Ethics Committees of all participating institutions (No. 100910001). The JECS is conducted in accordance with the Declaration of Helsinki and Ethical Guidelines for Medical and Biological Research Involving Human Subjects in Japan. Written informed consent was obtained from all participants involved in the study.

**Figure 1:**
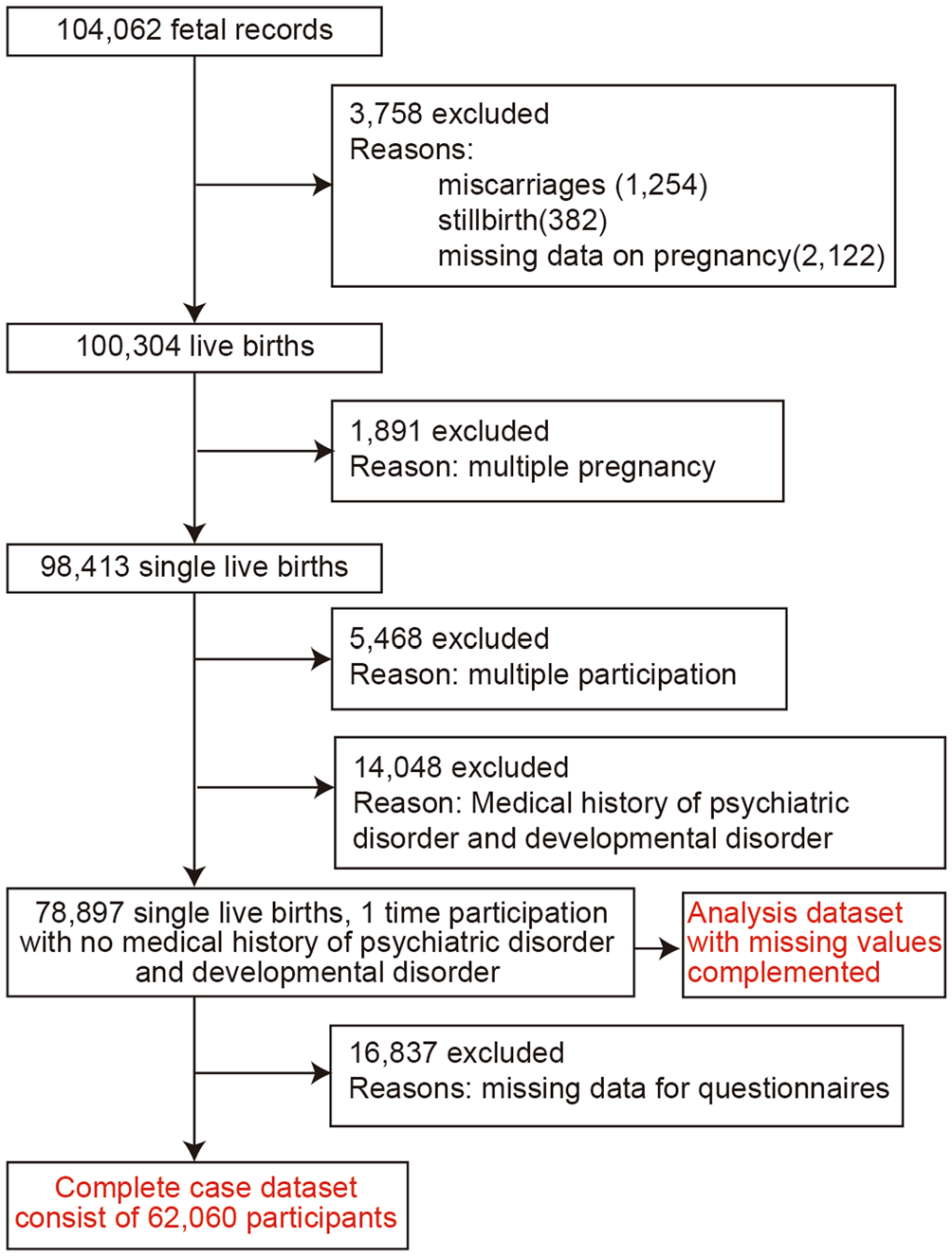
Flow diagram for selection of study participants.

In this study, we used a JECS dataset (jecs-ta-20190930) released in October 2019. The dataset does not contain any identifying patient information. In total, 104,062 fetal records were registered in 19 prefectures of Japan (Aichi, Chiba, Fukushima, Fukuoka, Hokkaido, Hyogo, Kanagawa, Kochi, Kumamoto, Kyoto, Miyagi, Miyazaki, Nagano, Okinawa, Osaka, Shiga, Toyama, Tottori, and Yamanashi) between January 2011 and March 2014. We excluded 3758 women because of miscarriage, stillbirth, or missing data on pregnancy, 1891 women with multiple birth, 5468 women who participated in the JECS multiple times, and 14,048 women because of a medical history of psychological disorders (depression, dysautonomia, anxiety disorder, schizophrenia, epilepsy, migraine, and other disorders) or developmental disorders (attention-deficit/hyperactivity disorder, learning disability, autism, and other disorders). Moreover, we excluded 16,451 women who did not complete the questionnaire. After exclusion, we analyzed complete data from 62,446 women (Fig. 1). We issued a questionnaire to each participant during their first trimester of pregnancy, either in person when the participant was recruited or by post, after explaining the purpose and practicalities of the study. Follow-up questionnaires were issued in person or by post during the second or third trimester, shortly after childbirth, and at 1 month postpartum during scheduled in-patient hospital checkups.

### Assessment of PPD

We used the Japanese version of the Edinburgh Postnatal Depression Scale (EPDS) to assess PPD. The EPDS is used worldwide for screening PPD. Data were collected from participants 1 month after delivery. The EPDS comprises 10 items, and responses are given to each item using a four-point scale (from 0 to 3). Higher scores indicate more severe depressive symptoms^23^. An optimal cut-off score of 8/9 has been reported for Japanese women at 1 month after delivery (75% sensitivity and 93% specificity)^24^. Therefore, we defined PPD at 1 month after delivery as an EPDS score ≥ 9.

### Assessment of weight-loss behavior

To assess weight-loss behavior during the year before the current pregnancy, a dichotomous questionnaire was administered to participants during their second or third trimester of pregnancy. Participants were asked: Did you engage in any of the following weight-loss behaviors during the year before current pregnancy? Multiple responses were possible. We defined women as using a weight-loss method if they checked at least one of the following weight-loss methods: eating two-thirds as much as usual or less, avoiding snacking and midnight snacking, eating specific diet foods, taking a diet pill, vomiting after eating, smoking cigarettes, and engaging in exercise. Participants answered yes or no for each item.

### Calculation of weight-loss score

To investigate the association between multiple weight-loss behaviors and PPD, we defined a weighted weight-loss method score. In a previous paper, weight-loss behaviors were classified as healthy and unhealthy weight-loss behavior^10^. Following that paper, we defined three types of weight-loss methods: we defined engaging in exercise and avoiding snacking and midnight snacking as weight-loss methods that were healthy; eating specific diet foods and eating two-thirds as much as usual or less as unhealthy weight-loss methods; taking a diet pill, vomiting after eating, and smoking cigarettes as extremely unhealthy weight-loss methods. In determining weight-loss method scores, we focused on the number of weight-loss method used and unhealthy weight-loss behaviors. To assess an excess degree of weight-loss method use, the weight-loss method score was calculated as the total value, with 1 point for healthy weight-loss methods, 2 points for unhealthy weight loss methods, and 3 points for extremely unhealthy weight-loss methods. The total score indicated the degree of weight-loss method use.

### Other confounding factors

Confounding factors comprised the following variables, obtained from the medical records: pre-pregnancy BMI (≤ 18.5, 18.5–24.9, ≥ 25 kg/m^2^), age of the mother (≤ 19, 20–24, 25–29, 30–34, 35–39, or ≥ 40 years), child’s sex (male or female), mode of delivery (spontaneous delivery, induced delivery, vacuum extraction/forceps delivery, or planned cesarean delivery/emergency cesarean delivery), number of previous deliveries (nullipara or multipara), diseases in the child currently under treatment (yes or no), how the mother became pregnant this time (spontaneously, ovulation induction through medication, artificial insemination in vitro fertilization, intracytoplasmic sperm injection, fresh embryo transfer, frozen embryo transfer, blastocyst transfer), recurrent miscarriage (yes or no), and gestational weight gain (normal weight gain or abnormal weight gain; abnormal weight gain was defined following the Optimal Weight Gain Chart for Pregnancy published by the Ministry of Health, Labour and Welfare in 2006^25^). Covariates comprised the following variables, obtained using self-administered questionnaires completed by participants: current marital status (married, single, divorced/widowed), feelings when learning about the pregnancy (very happy, surprised but happy, surprised and confused, upset, did not have any specific or other feelings), experience of any stressful events during the past year (yes or no), having someone who can be counted on to provide emotional support (never, rarely, some of the time, most of the time, or all of the time), alcohol intake (never, past, or current), highest level of education (junior high school/high school, technical junior college/technical or vocational college/associate degree, or bachelor’s degree/graduate degree), annual household income (< 4 million JPY, 4 to < 6 million JPY, or ≥ 6 million JPY), receiving emotional abuse from husband/romantic partner during current pregnancy (yes or no), receiving physical abuse from husband/romantic partner leading to injury during current pregnancy (yes or no).

Women’s antenatal psychological distress was assessed using the Japanese version of the Kessler 6 scale (K6)^26^. The data were collected during the first trimester. The K6 comprises six items, with higher scores indicating more severe psychological distress. An optimal cut-off score of 12/13 has been reported^26,27^; therefore, we defined antenatal psychological distress as a K6 score ≥ 13.

### Statistical analysis

The crude odds ratio and adjusted odds ratio (aOR) were calculated in binomial logistic regression analyses. Briefly, to calculate the crude odds ratio, a model was constructed using PPD as the dependent variable and the weight-loss method or weight-loss method score as independent variables. To calculate the aOR, a model was constructed using PPD as the dependent variable and the weight-loss method or score and confounding factors that were significantly associated with PPD (according to the Mann–Whitney U test or chi-square test) as independent variables. Significance was defined as p < 0.05, and 95% confidence intervals (CIs) were calculated. As a sensitivity analysis to address the influence of missing values, we used a multiple imputation method. Briefly, 100 simulated datasets were generated in a multiple imputation method using the R mice package^28^. Each simulated dataset was analyzed in logistic regression analysis; each estimation was combined with pooling rules^29^. To assess the presence of collinearity, we calculated the variance inflation factor (VIF) and checked for values < 10. The VIF values were as follows: age of the mother: 1.39; child's sex: 1.00; mode of delivery: 1.09; number of previous deliveries: 1.21; marital status: 1.11; feeling when learning about pregnancy: 1.11; stressful events: 1.05; emotional support: 1.06; highest level of education: 1.21; annual household income: 1.18; emotional abuse from partner: 1.21; physical abuse from partner: 1.13; BMI before pregnancy: 1.07; diseases in child currently under treatment: 1.00; how the mother became pregnant this time: 1.10; recurrent miscarriage: 1.01; psychological distress: 1.02; and gestational weight gain: 1.06. All analyses and graphs were done using R version 4.1.1^30^.

### Ethics approval

The JECS protocol was reviewed and approved by the Ministry of the Environment’s Institutional Review Board on Epidemiological Studies and the Ethics Committees of all participating institutions. The JECS was conducted in accordance with the Declaration of Helsinki and other nationally valid regulations and guidelines. Written informed consent to take part in this survey was obtained from all participants.

## Results

Table 1 and Supplementary Table S1 show the main characteristics of the 62,060 participants according to EPDS scores, and the complete characteristics of 78,897 participants with missing values, respectively. At 1 month after delivery, 7464 (12%) women had PPD (EPDS ≥ 9) and 54,596 (88%) women did not have PPD.

**Table 1:** Characteristics of participants according to EPDS score (N = 62,060).

Characteristics	No PPD (EPDS < 9) n = 54,596		PPD (EPDS ≥ 9) n = 7464		p-value
	n	%	n	%	
Women's factors including socio-economic status					
BMI in first trimester (kg/m^2^)					
< 18.5	8542	13.8	1184	1.9	< 0.001^b^
18.5–24.9	40,538	65.3	5407	8.7	
≥ 25	5516	8.9	873	1.4	
Age of mother (years)					
≤ 19	270	0.4	70	0.1	< 0.001^b^
20–24	4032	6.5	841	1.4	
25–29	14,710	23.7	2210	3.6	
30–34	19,928	32.1	2502	4.0	
35–39	13,027	21.0	1516	2.4	
≥ 40	2629	4.2	325	0.5	
Alcohol intake					
Never	19,182	30.9	2451	3.9	< 0.001^b^
Previous	29,651	47.8	4320	7.0	
Current	5763	9.3	693	1.1	
Marital status					
Married	52,790	85.1	6995	11.3	< 0.001^b^
Single	1463	2.4	376	0.6	
Divorced, widowed	343	0.6	93	0.1	
The number of people who provide emotional supports					
Median individuals	5		4		< 0.001^a^
Highest level of education					
Junior high school, high school	18,037	29.1	3055	4.9	< 0.001^b^
Technical junior college, technical/vocational college, associate degree	23,815	38.4	2994	4.8	
Bachelor’s degree, graduate degree	12,744	20.5	1415	2.3	
Annual household income					
< 4 million JPY	20,708	33.4	3469	5.6	< 0.001^b^
4 to < 6 million JPY	18,429	29.7	2316	3.7	
≥ 6 million JPY	15,459	24.9	1679	2.7	
The factors related pregnancy					
Child's sex					
Male	27,923	45.0	3906	6.3	0.420^b^
Female	26,673	43.0	3558	5.7	
Mode of delivery					
Spontaneous delivery	31,727	51.1	4078	6.6	< 0.001^b^
Induced delivery	9651	15.6	1441	2.3	
Vacuum extraction, forceps delivery	3262	5.3	517	0.8	
Cesarean delivery	9956	16.0	1428	2.3	
Previous deliveries					
Nullipara	21,808	35.1	4008	6.5	< 0.001^b^
Multipara	32,788	52.8	3456	5.6	
Feelings when learning of pregnancy					
Very happy	37,059	59.7	4349	7.0	< 0.001^b^
Surprised but happy	13,371	21.5	2122	3.4	
Surprised and confused	3274	5.3	721	1.2	
Upset	215	0.3	89	0.1	
No specific feelings	209	0.3	55	0.1	
Other feelings	468	0.8	128	0.2	
Stressful events during pregnancy					
No	32,815	52.9	3315	5.3	< 0.001^b^
Yes	21,781	35.1	4149	6.7	
Disease in child currently under treatment					
No	48,657	78.4	6540	10.5	< 0.001^b^
Yes	5939	9.6	924	1.5	
How the mother became pregnant this time					
Spontaneously	50,869	82.0	6963	11.2	0.087^b^
Ovulation induction through medication	1488	2.4	196	0.3	
AIH	498	0.8	85	0.1	
IVF	922	1.5	121	0.2	
ICSI	439	0.7	54	0.1	
Fresh embryo transfer	24	0.0	0	0.0	
Frozen embryo transfer	303	0.5	33	0.1	
Blastocyst transfer	53	0.1	12	0.0	
Recurrent miscarriage					
No	54,015	87.0	7386	11.9	0.521^b^
Yes	581	0.9	78	0.1	
Gestational weight gain					
Normal weight gain	28,906	46.6	3817	6.2	0.003^b^
Abnormal weight gain	25,690	41.4	3647	5.9	
Emotional abuse from partner during pregnancy					
No	48,452	78.1	5626	9.1	< 0.001^b^
Yes	6144	9.9	1837	3.0	
Physical abuse from partner during pregnancy					
No	54,160	87.3	7254	11.7	< 0.001^b^
Yes	436	0.7	210	0.3	
Psychological distress					
K6 < 13	53,769	86.6	6693	10.8	< 0.001^b^
K6 ≥ 13	827	1.3	771	1.2	
Weight-loss methods before pregnancy					
Eating two-thirds as much as usual or less					
No	47,853	77.1	6112	9.8	< 0.001^b^
Yes	6743	10.9	1352	2.2	
Avoiding snacks and midnight snacks					
No	34,079	54.9	4403	7.1	< 0.001^b^
Yes	20,517	33.1	3061	4.9	
Eating specific diet foods					
No	49,484	79.7	6464	10.4	< 0.001^b^
Yes	5112	8.2	1000	1.6	
Taking a diet pill					
No	53,621	86.4	7214	11.6	< 0.001^b^
Yes	975	1.6	250	0.4	
Vomiting after eating					
No	53,958	86.9	7251	11.7	< 0.001^b^
Yes	638	1.0	213	0.3	
Smoking cigarettes					
No	52,608	84.8	6894	11.1	< 0.001^b^
Yes	1988	3.2	570	0.9	
Exercise					
No	37,543	60.5	4770	7.7	< 0.001^b^
Yes	17,053	27.5	2694	4.3	

*EPDS* Edinburgh Postnatal Depression Scale, *PPD* postpartum depression, *BMI* body mass index, *AIH* artificial insemination with husband’s sperm, *IVF* in vitro fertilization, *ICSI* intracytoplasmic sperm injection, *K6* Kessler 6-Item Psychological Distress Scale.

^a^Mann–Whitney* U* test.

^b^Chi-square test.

To elucidate the association between using at least one weight-loss method and PPD, we performed logistic regression analysis (Table 2). We defined the reference group as women who did not engage in any weight-loss behaviors. Because PPD is associated with depression during pregnancy, we stratified psychological distress during the second or third trimester. In women who did not have psychological distress during the second or third trimester (K6 < 13), the aOR for PPD was 1.318 (95% CI: 1.246–1.394) in those using at least one weight-loss method. In women with psychological distress during the second or third trimester (K6 ≥ 13), the aOR for PPD was 1.250 (CI: 0.999–1.565) in those using at least one weight-loss method. Although there was no significant association between PPD and using at least one weight-loss method in women with psychological distress during pregnancy, there was a positive trend between PPD and weight-loss behaviors before pregnancy because of the 95% CI was 0.999 to 1.565.

**Table 2:** Odds ratio (95% CI) of PPD according to use of weight-loss methods, after excluding participants with a medical history of psychiatric disorders.

	*N*	aOR^a^	Lower 95% CI	Higher 95% CI	p-value
K6 score < 13					
No weight-loss method	39,774	1.000			
Using at least one weight-loss method	6171	1.318	1.246	1.394	< 0.001
K6 score ≥ 13					
No weight-loss method	532	1.000			
Using at least one weight-loss method	1066	1.250	0.999	1.565	0.052

*Cl* confidence interval, *aOR* adjusted odds ratio, *PPD* postpartum depression, *K6* Kessler 6-Item Psychological Distress Scale, *BMI* body mass index.

^a^Adjusted for age of the mother, sex, mode of delivery, number of previous deliveries, marital status, feelings when learning about pregnancy, stressful events, emotional support, highest level of education, annual household income, emotional abuse from partner, physical abuse from partner, BMI in first trimester, disease in the child currently under treatment, how the mother became pregnant this time, recurrent miscarriage, and gestational weight gain.

Next, there is the possibility that the impact on PPD was varied depending on the type of weight-loss behavior before pregnancy. To elucidate this, a logistic regression analysis was performed between PPD and each weight-loss method. In univariate logistic regression analysis, there was significant association between each weight-loss methods and PPD (Table 3). Even if we performed multivariate logistic regression analysis using potential confounding factors, we could find the association between each weight-loss methods and PPD (Table 3). These results indicated that all weight-loss methods before pregnancy were significantly associated with PPD.

**Table 3:** Odds ratio (95% CI) of PPD according to each weight-loss method.

Weight-loss methods	*N*	Crude				Adjusted^a^			
		OR	Lower 95% CI	Higher 95% CI		OR	Lower 95% CI		Higher 95% CI
Eating two-thirds as much as usual or less									
No	53,965	1.000				1.000			
Yes	8095	1.570	1.472	1.673		1.324	1.236		1.418
Avoiding snacking and midnight snacks									
No	38,482	1.000				1.000			
Yes	23,578	1.155	1.099	1.213		1.101	1.045		1.160
Eating specific diet foods									
No	55,948	1.000				1.000			
Yes	6112	1.498	1.392	1.610		1.250	1.156		1.351
Taking diet pills									
No	60,835	1.000				1.000			
Yes	1225	1.906	1.652	2.191		1.308	1.122		1.520
Vomiting after eating									
No	61,209	1.000				1.000			
Yes	851	2.484	2.119	2.901		1.743	1.465		2.065
Smoking cigarettes									
No	59,502	1.000				1.000			
Yes	2558	2.188	1.985	2.408		1.432	1.287		1.591
Exercise									
No	42,313	1.000				1.000			
Yes	19,747	1.243	1.182	1.308		1.132	1.072		1.194

*EPDS* Edinburgh Postnatal Depression Scale, *PPD* postpartum, depression, *CI* confidence interval, *OR* odds ratio.

^a^Adjusted for age of mother, sex, mode of delivery, number of previous deliveries, marital status, feelings when learning about pregnancy, stressful events, emotional support, highest level of education, annual household income, emotional abuse from partner, physical abuse from partner, BMI before pregnancy, disease in child currently under treatment, how the mother became pregnant this time, recurrent miscarriage, psychological distress, and gestational weight gain.

**Figure 2:**
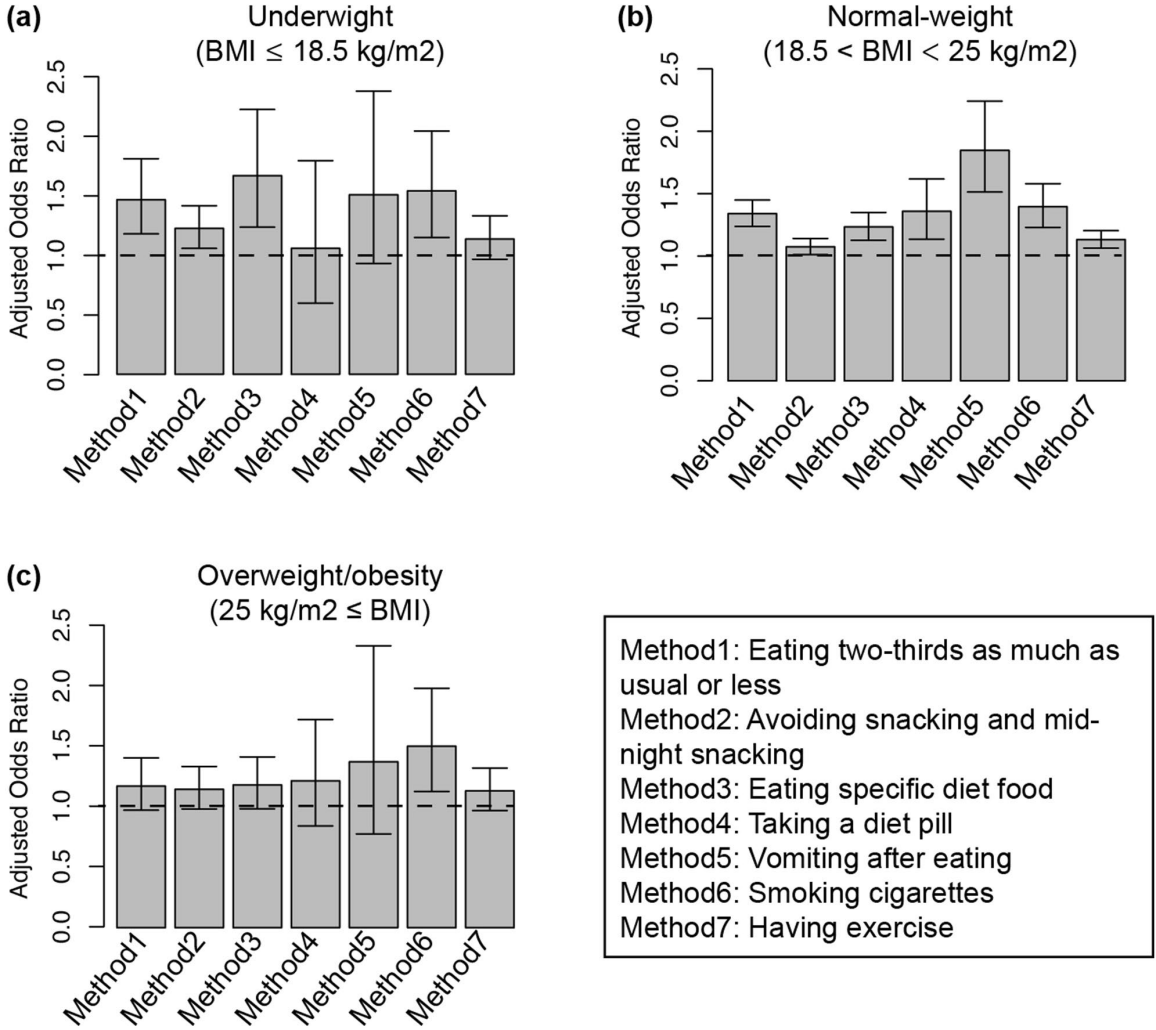
Association between weight-loss method use before pregnancy and PPD in BMI-stratified analysis for **(a)** underweight women (N = 9726), **(b)** normal-weight women (N = 45,945), and **(c)** overweight/obesity women (N = 6389). Adjusted odds ratio and 95% confidence interval was calculated by multivariate logistic regression analysis. Error bar represents 95% confidence interval. Dotted line indicates the reference category (women who did not engage in any weight-loss behaviors). Adjusted for age of the mother, child’s sex, mode of delivery, number of previous deliveries, marital status, feelings when learning about the pregnancy, stressful events, emotional support, highest level of education, annual household income, emotional abuse from partner, physical abuse from partner, diseases in the child currently under treatment, how the mother became pregnant this time, recurrent miscarriage, psychological distress, and gestational weight gain. Fully information such as adjusted odds ratio, 95% confidence interval and number of women in each groups were indicated supplemental Table 2. *PPD* postpartum depression, *BMI* body mass index.

Although we adjusted for BMI before pregnancy in logistic regression analysis, the association between PPD and use of weight-loss method may differ by pre-pregnancy BMI. To clarify this, we performed logistic regression analysis after stratification by pre-pregnancy BMI (Fig. 2, Supplementary Table S2). We separated out three datasets from the original dataset according to BMI before pregnancy (underweight: BMI < 18.5 kg/m^2^; normal weight: BMI 18.5 to < 25 kg/m^2^; overweight or obese: BMI ≥ 25 kg/m^2^). Interestingly, we found a significant association in underweight women between PPD and eating two-thirds as much as usual or less, avoiding snacking and midnight snacking, eating specific diet foods, and smoking cigarettes (Fig. 2a, Supplementary Table S2). PPD was not associated with vomiting after eating or engaging in exercise among underweight women. In women with normal BMI, all weight-loss methods were associated with PPD (Fig. 2b, Supplementary Table S2). In overweight or obese women, we found a significant association between PPD and smoking cigarettes only; there was no association between PPD and other methods (Fig. 2c, Supplementary Table S2). These results indicated that the association between weight-loss methods and PPD varied according to BMI before pregnancy.

**Figure 3:**
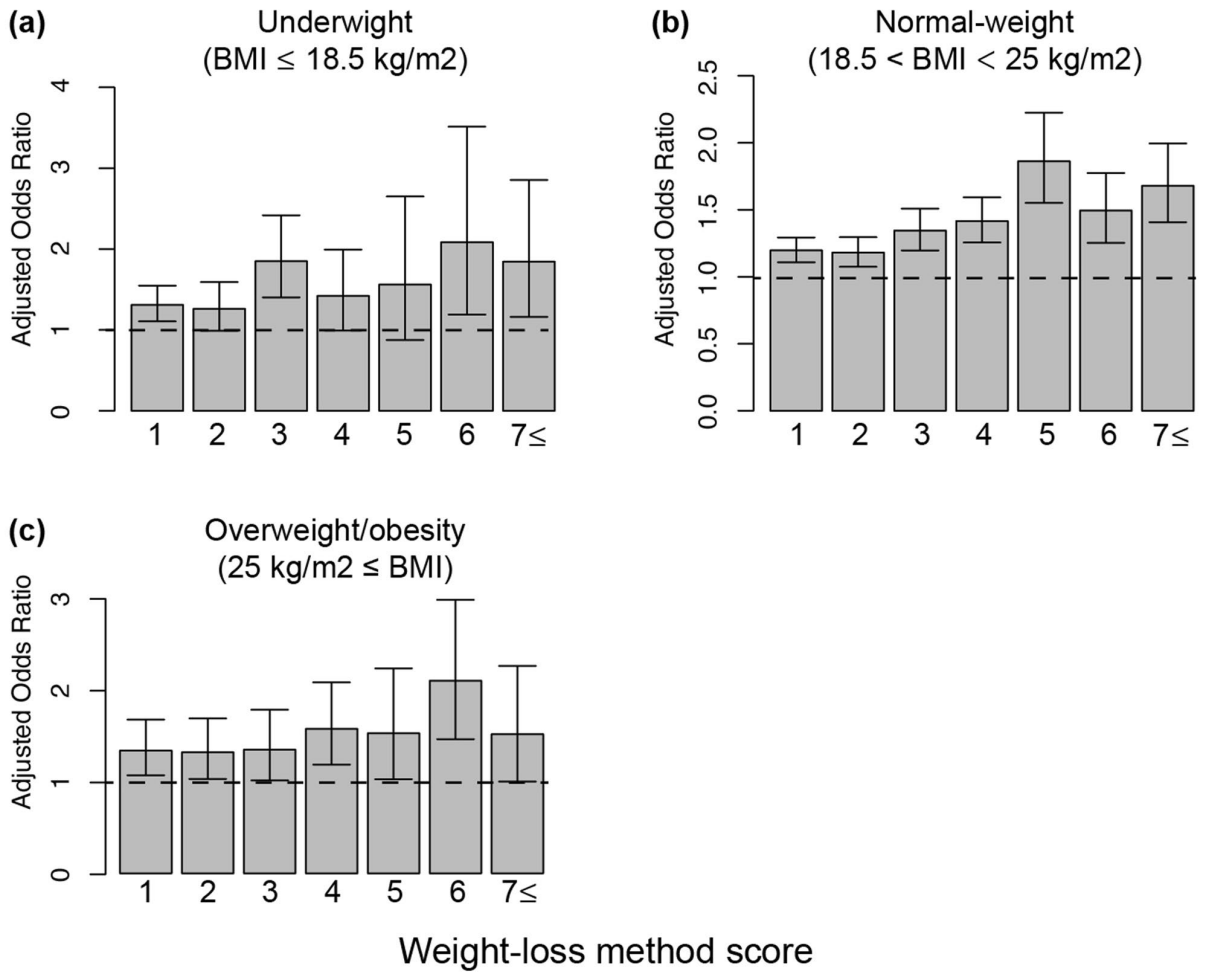
Risk of postpartum depression was increased with increased weight-loss method score in a dependent manner. **(a)** Association between weight-loss method score before pregnancy and PPD in underweight women (N = 9726). **(b)** Association between weight-loss method score before pregnancy and PPD in normal-weight women (N = 45,945) **(c)** Association between weight-loss method score before pregnancy and PPD in overweight/obesity women (N = 6389). Adjusted odds ratio and 95% confidence interval was calculated by multivariate logistic regression analysis. Error bar represents 95% confidence interval. Dotted line indicates the reference category (women who did not engage in any weight-loss behaviors). Adjusted for age of the mother, child’s sex, mode of delivery, number of previous deliveries, marital status, feelings when learning about the pregnancy, stressful events, emotional support, highest level of education, annual household income, emotional abuse from partner, physical abuse from partner, diseases in the child currently under treatment, how the mother became pregnant this time, recurrent miscarriage, psychological distress, and gestational weight gain. Fully information such as adjusted odds ratio, 95% confidence interval and number of women in each groups were indicated supplemental Table 3. *PPD* postpartum depression, *BMI* body mass index

To elucidate the association between using multiple weight-loss methods and PPD, we calculated the weight-loss method score. The weight-loss method score indicates the weighted degree of weight-loss method use and a weight-loss score of 0 indicates no weight-loss method used. To investigate the effect of weight-loss score on PPD, we included a dummy variable for weight-loss score and performed logistic regression analysis. Although we could observed the significant association between weight-loss score and PPD compared with the women with a weight-loss score of 0, the aORs were fluctuated in all BMI group (Fig. 3, Supplementary Table S3). In underweight women, the aOR was increased, with a score of 3. A weight-loss score of 3 indicated use of healthy weight-loss method and one unhealthy weight-loss method. Therefore, using at least one unhealthy weight-loss method was associated with PPD at 1 month after delivery. In normal-weight women who engaged in multiple weight-loss behaviors, weight-loss score was significantly associated with an increased risk of PPD at 1 month after delivery. From score 1 point to 5 points, the ORs increased in a score-dependent manner. In overweight/obese women, the weight-loss score of 4, which indicated use of multiple healthy weight-loss method and at least one unhealthy weight-loss methods or two unhealthy weight-loss methods, was significantly associated with PPD. These results showed that excessive use of multiple weight-loss methods and unhealthy weight-loss methods before pregnancy were associated with PPD at 1 month after delivery.

We included only participants with a complete dataset and excluded those with missing data (Fig. 1). However, there was a possible effect of missing values on our results. To clarify this, we performed logistic regression analysis using a simulated dataset, with missing values generated using multiple imputation methods (Supplementary Tables S4 and S5, Supplementary Figs. S1 and S2). The results were the same using the simulated dataset; thus, missing values did not affect the results in this study.

## Discussion

There are no studies on the association between PPD and weight-loss behaviors before pregnancy in Japanese women. We performed logistic regression analysis using PPD and weight-loss behaviors and using JECS data. The results revealed a significant association between weight-loss behaviors before pregnancy and PPD. The association between using weight-loss methods and PPD varied according to BMI before pregnancy. Using multiple weight-loss methods were significantly associated with the increasing the risk of PPD. These results may indicated that weight-loss behaviors increased the PPD risk for Japanese women. It is important to educate women considering pregnancy about the dangers of excessive or unhealthy weight-loss behaviors.

In general, it is reported that women should begin a pregnancy with a healthy body weight because of the increased risk of pregnancy complications^31^. In overweight or obese women, it is recommended to reach a healthy weight before conceiving, such as with counseling on diet and physical activity^31^. However, our results indicated a significant association between weight-loss behaviors before pregnancy and PPD (Table 2). This result seemed to contradict the statement about weight-loss behavior before pregnancy to reach a healthy weight. However, our results also indicated the association between PPD and weight-loss behavior was varied in different BMI groups before pregnancy (Fig. 2, Supplementary Table S2). Interestingly, there was no association between weight-loss behavior and PPD in overweight/obese women (Fig. 2c, Supplementary Table S2). We noted that only smoking cigarettes was associated with PPD (Fig. 2c, Supplementary Table S2). Thus, our results underscore the importance of weight-loss behavior before pregnancy.

In normal-weight women, unhealthy weight-loss behaviors such as taking a diet pill, smoking cigarettes, and vomiting after eating were associated with PPD, and there was an association between smoking cigarettes and PPD in underweight women. Because smoking cigarettes is associated with PPD but not the goal of weight loss, this result was consistent with those of previous studies^20^. Other extremely unhealthy weight-loss behaviors, such as taking diet pills and vomiting after eating, were associated with PPD in normal-weight women. Although the precise mechanism is unknown, these results indicated that eating behaviors related to weight loss before pregnancy may be associated with PPD, at least in normal-weight women. Therefore, to prevent PPD, it is important to raise awareness among women about the risk of using weight-loss methods according to their pre-pregnancy BMI.

It was previously reported that unhealthy weight-loss behaviors were associated with psychological disorders^9,10^. Eating disorders, such as binge eating disorder and bulimia nervosa, are associated with PPD^32^. Although vomiting itself may not be related to losing weight or to eating disorders, some studies have found a positive association between vomiting owing to nausea during pregnancy and PPD^33,34^. However, there are no studies on a direct association between vomiting to lose weight before pregnancy and PPD. In this study, vomiting after eating was associated with PPD in women with normal BMI but not in underweight and overweight/obese women (Table 3, Fig. 2, Supplementary Table S2). Because eating disorders occur intercurrently with other psychological disorders, we excluded participants with a medical history of psychological disorders and developmental disorders from the data analysis; this group may have included women with a history of eating disorders. Thus, our results indicated that extreme weight-loss behaviors, but not those owing to eating disorders, may be directly associated with PPD in normal-weight women.

Generally, exercise is a protective factor for mental health. A previous study reported that physical activity during pregnancy reduced the risk of PPD^35^. Surprisingly, however, weight-loss behaviors that were not unhealthy, such as avoiding snacking and midnight snacking and engaging in exercise, were also associated with PPD in normal-weight women (Fig. 2b, Supplementary Table S2). These results are inconsistent with those of previous studies. We queried participants regarding whether they engaged in exercise as a weight-loss method. Because approximately 90% of Japanese women in their 20s and 30s are underweight or normal weight, most Japanese women are either thin or within the normal weight range^1^. In this study, 55,671 (89.7%) pregnant women were not obese before pregnancy (Table 1). Interestingly, exercise was not associated with PPD in overweight/obese women (Fig. 2c, Supplementary Table S2). This indicates that despite most women in Japan not needing to lose weight, many engage in weight-loss behaviors, such as excessive exercise and excessive limitation of nutrient intake, and these excessive behaviors may be associated with PPD. Additionally, the desire to be thin is common among many women. The desire to lose weight may be associated with the risk of PPD, but further studies are needed to investigate this relationship.

The current findings revealed an association between weighted weight-loss method scores and the risk of PPD in women with any BMI (Fig. 3, Supplementary Table S3). This was the first study to report that the number of weight-loss methods used affects PPD, with a higher PPD risk associated with an increasing number of methods used in normal-weight women. Women with weight-loss scores of more than 3 points engaged in multiple weight-loss behaviors, such as trying both healthy and unhealthy ways to lose weight. In normal-weight women, engaging in healthy as well as unhealthy weight-loss behavior associated with increase the risk of PPD, as compared with engaging in one weight-loss behavior (Fig. 3b, Supplementary Table S3). Because women who undertake weight loss using multiple methods often engage in extreme weight-loss behaviors, they may experience physical and psychological problems, such as poor nutrition and eating disorders. Additionally, women who use multiple weight-loss methods may be obsessed with their body image and feel the need to lose weight. Such physical and psychological problems before pregnancy may lead to PPD after delivery in women with any BMI. Interestingly, the associations between weight-loss methods and PPD were varied by pre-pregnancy BMI (Figs. 2, 3, Supplementary Tables S2, S3). In overweight/obese women, weight-loss behavior except smoking cigarettes was not associated with PPD (Fig. 2c, Supplementary Table S2). These results may indicate that the desire to be thin in normal and skinny women is stronger than that in overweight/obese women. The desire to be thin may be associated with PPD in women with normal and low BMI values. Therefore, awareness among women about the risk of using extreme weight-loss methods is important to prevent PPD and psychological problems.

There are six limitations in this study. First, weight-loss behavior was assessed using dichotomous-variable questions. Therefore, we could not obtain information regarding the frequency, degree, and time frame of weight-loss behaviors using the questionnaires. Nevertheless, these questions have been used in several studies^10,36,37^. Therefore, we believe that the use of dichotomous-variable questions did not pose a problem in this study. To reveal further details regarding weight-loss behaviors before pregnancy, further studies are warranted. Second, we collected information about weight-loss behavior before pregnancy when participants were in their second or third trimester of pregnancy, which could introduce recall bias. For example, women who were not overly concerned about their body image may report engaging in no weight-loss behaviors even if weight-loss methods were used. To remove any bias, objective indicators of weight-loss methods should be used to clarify this. Third, we could not obtain BMI data at the time that our participants began to use weight-loss methods. Therefore, we could not include the success or failure of a weight-loss method as adjustment variables. However, we observed a significant association between weight-loss behavior and PPD, even with stratification of BMI before pregnancy. Because approximately 90% of Japanese women in their 20s and 30s are within the normal weight range or below, there may be an association between PPD and extreme or excessive weight-loss behavior. Further studies are required to clarify the associations among PPD, weight-loss behavior, and BMI in detail. Fourth, we could not completely eliminate the effect of eating disorders in the analysis. We excluded participants with psychological disorders and developmental disorders from the data analysis; however, we may not have eliminated participants with borderline eating disorders from the analysis. To clarify the precise association between eating disorders and PPD, further investigation is needed using questionnaires on eating disorders, such as the Eating Attitudes Test-26. Fifth, we could not measure the desire to be thin among women in this study. This desire may be associated with PPD. To clarify the precise association, further research is needed. Sixth, we used weighted weight-loss scores. There was a fluctuation in the aOR between PPD and weighted weight-loss scores, especially in underweight women (Fig. 3, Supplementary Table S3). This is an important limitation of this score. Because we calculated the total score in the analysis, unhealthy weight-loss behavior and multiple healthy weight-loss methods (e.g., a score of 3 included women who used three healthy weight-loss methods and those who used one extremely unhealthy weight-loss method) were treated equally. However, it is unknown if these are actually equivalent. Thus, there might be a fluctuation in the aOR between PPD and weighted weight-loss scores. To elucidate this, new precisely validated weight-loss scores should be developed, and further studies are needed.

Despite these limitations, the strengths of the current study included the large sample size in Japan and prospective data collection. To the best of our knowledge, this study was the first to investigate the association between weight-loss behaviors in the year before pregnancy and the risk of PPD in a large sample of women throughout Japan. Our findings provide further evidence regarding the health risks of unhealthy weight-loss behavior among Japanese women.

In conclusion, the current study revealed that using weight-loss methods in the year before pregnancy was associated with an increased risk of PPD in Japanese women. Many Japanese women have a desire to lose weight and may have difficulty avoiding weight-loss behaviors. The media have created an image in society that women must lose weight and be thin. However, our findings highlight the risks associated with unhealthy and excessive weight-loss behavior among Japanese women who plan to become pregnant in the near future. It is important to educate Japanese women, particularly those planning pregnancy, regarding the association between weight-loss behaviors and mental health.

## Supplementary Information

Supplementary Information.

## Data Availability

The data in this study are unsuitable for public dissemination owing to ethical restrictions and the legal framework in Japan. This is prohibited by the Act on the Protection of Personal Information (Act No. 57 of 30 May 2003, amended on 9 September 2015) to publicly deposit data containing personal information. The Ethical Guidelines for Medical and Health Research Involving Human Subjects, enforced by the Japan Ministry of Education, Culture, Sports, Science and Technology and the Ministry of Health, Labour and Welfare, also restrict open sharing of the epidemiologic data. All inquiries about access to the data should be sent to: jecs-en@nies.go.jp. The person responsible for handling enquiries is Dr Shoji F. Nakayama, JECS Programme Office, National Institute for Environmental Studies.
