# Supplementary material for: Weight-loss behaviors before pregnancy associate with increased risk of postpartum depression from the Japan Environment and Children’s Study
Source: Sci Rep. 2023 May 5;13:7363. doi: 10.1038/s41598-023-34547-4 (PMC10163226; doi:10.1038/s41598-023-34547-4)

*Supplementary information for*

**Weight-loss behaviors before pregnancy associate with increased risk of postpartum depression from the Japan Environment and Children’s Study**

Saki Taniguchi^1^, Toshio Masumoto^2^*, Youichi Kurozawa^2^

1 Tottori Regional Center of The Japan Environment and Children’s Study, Faculty of Medicine, Tottori University, Tottori, Japan

2 Division of Health Administration and Promotion, Department of Social Medicine, Faculty of Medicine, Tottori University, Tottori, Japan

* Correspondence: Toshio Masumoto (email: [tmasumoto@tottori-u.ac.jp](mailto:tmasumoto@tottori-u.ac.jp))

**Supplemental Table 1.** Complete Characteristics of participants according to EPDS score (N = 78,897).

|  |  |  | No PPD (EPDS < 9) | |  | With PPD (EPDS ≥9) | |  | Not Ansewed EPDS | |
| --- | --- | --- | --- | --- | --- | --- | --- | --- | --- | --- |
| Characteristics |  |  | n | Proportion(%) |  | n | Proportion(%) |  | n | Proportion(%) |
|  | Total |  | 66859 | 84.7 |  | 9546 | 12.1 |  | 2492 | 3.2 |
| *Women's factors including socio-economic status* | |  |  |  |  |  |  |  |  |  |
|  | BMI in first trimester (kg/m2) | <18.5 | 10637 | 13.5 |  | 1597 | 2.0 |  | 455 | 0.6 |
|  |  | 18.5-24.9 | 49457 | 62.7 |  | 6842 | 8.7 |  | 1730 | 2.2 |
|  |  | 25< | 6736 | 8.5 |  | 1104 | 1.4 |  | 303 | 0.4 |
|  |  | NA | 29 | 0.0 |  | 3 | 0.0 |  | 4 | 0.0 |
|  | Age of mother (years) | ≤19 | 498 | 0.6 |  | 141 | 0.2 |  | 68 | 0.1 |
|  |  | 20-24 | 5409 | 6.9 |  | 1200 | 1.5 |  | 378 | 0.5 |
|  |  | 25-29 | 18115 | 23.0 |  | 2821 | 3.6 |  | 668 | 0.8 |
|  |  | 30-34 | 24037 | 30.5 |  | 3123 | 4.0 |  | 763 | 1.0 |
|  |  | 35-39 | 15621 | 19.8 |  | 1864 | 2.4 |  | 483 | 0.6 |
|  |  | ≥40 | 3174 | 4.0 |  | 396 | 0.5 |  | 132 | 0.2 |
|  |  | NA | 5 | 0.0 |  | 1 | 0.0 |  | 0 | 0.0 |
|  | Alcohol intake | Never | 23306 | 29.5 |  | 3154 | 4.0 |  | 853 | 1.1 |
|  |  | Stopped | 36390 | 46.1 |  | 5507 | 7.0 |  | 1389 | 1.8 |
|  |  | Continue | 6876 | 8.7 |  | 845 | 1.1 |  | 220 | 0.3 |
|  |  | NA | 287 | 0.4 |  | 40 | 0.1 |  | 30 | 0.0 |
|  | Marital status | Married | 64050 | 81.2 |  | 8793 | 11.1 |  | 2277 | 2.9 |
|  |  | Single | 2125 | 2.7 |  | 550 | 0.7 |  | 154 | 0.2 |
|  |  | Divorced, widowed | 441 | 0.6 |  | 131 | 0.2 |  | 34 | 0.0 |
|  |  | NA | 243 | 0.3 |  | 72 | 0.1 |  | 27 | 0.0 |
|  | The number of people who provide emotional supports | Median individuals | 5 |  |  | 4 |  |  | 5 |  |
|  | Highest level of education | Junior high school, high school | 22367 | 28.3 |  | 3973 | 5.0 |  | 979 | 1.2 |
|  |  | Technical junior college, technical/vocational college, associate degree | 28657 | 36.3 |  | 3723 | 4.7 |  | 779 | 1.0 |
|  |  | Bachelor’s degree, graduate | 15141 | 19.2 |  | 1731 | 2.2 |  | 383 | 0.5 |
|  |  | NA | 694 | 0.9 |  | 119 | 0.2 |  | 351 | 0.4 |
|  | Annual household income | <4 million JPY | 23509 | 29.8 |  | 4070 | 5.2 |  | 859 | 1.1 |
|  |  | 4 to <6 million JPY | 20894 | 26.5 |  | 2654 | 3.4 |  | 562 | 0.7 |
|  |  | ≥6 million JPY | 17618 | 22.3 |  | 1943 | 2.5 |  | 468 | 0.6 |
|  |  | NA | 4838 | 6.1 |  | 879 | 1.1 |  | 603 | 0.8 |
| *The factors related pregnancy* |  |  |  |  |  |  |  |  |  |  |
|  | Child's Sex | male | 34191 | 43.3 |  | 5057 | 6.4 |  | 1321 | 1.7 |
|  |  | Female | 32666 | 41.4 |  | 4489 | 5.7 |  | 1163 | 1.5 |
|  |  | Unknown | 0 | 0.0 |  | 0 | 0.0 |  | 3 | 0.0 |
|  |  | NA | 2 | 0.0 |  | 0 | 0.0 |  | 5 | 0.0 |
|  | Mode of delivery | Spontaneous delivery | 38627 | 49.0 |  | 5230 | 6.6 |  | 1314 | 1.7 |
|  |  | Induced delivery | 11796 | 15.0 |  | 1801 | 2.3 |  | 381 | 0.5 |
|  |  | Vacuum extraction, forceps delivery | 3909 | 5.0 |  | 666 | 0.8 |  | 111 | 0.1 |
|  |  | Cesarean delivery | 12240 | 15.5 |  | 1804 | 2.3 |  | 596 | 0.8 |
|  |  | NA | 287 | 0.4 |  | 45 | 0.1 |  | 90 | 0.1 |
|  | Number of previous deliveries | Nullipara | 26435 | 33.5 |  | 4995 | 6.3 |  | 1031 | 1.3 |
|  |  | Multipara | 38862 | 49.3 |  | 4228 | 5.4 |  | 1385 | 1.8 |
|  |  | NA | 1562 | 2.0 |  | 323 | 0.4 |  | 76 | 0.1 |
|  | Feelings when learning of pregnancy | Very happy | 45097 | 57.2 |  | 5469 | 6.9 |  | 1510 | 1.9 |
|  |  | Unexpected but happy | 16340 | 20.7 |  | 2713 | 3.4 |  | 674 | 0.9 |
|  |  | Unexpected and confused | 4002 | 5.1 |  | 951 | 1.2 |  | 217 | 0.3 |
|  |  | Upset | 267 | 0.3 |  | 115 | 0.1 |  | 22 | 0.0 |
|  |  | Did not have any specific feeling | 270 | 0.3 |  | 68 | 0.1 |  | 13 | 0.0 |
|  |  | Other feeling | 580 | 0.7 |  | 164 | 0.2 |  | 14 | 0.0 |
|  |  | NA | 303 | 0.4 |  | 66 | 0.1 |  | 42 | 0.1 |
|  | Stressful events during pregnancy | No | 39762 | 50.4 |  | 4193 | 5.3 |  | 1257 | 1.6 |
|  |  | Yes | 26295 | 33.3 |  | 5226 | 6.6 |  | 892 | 1.1 |
|  |  | NA | 802 | 1.0 |  | 127 | 0.2 |  | 343 | 0.4 |
|  |  | NA | 729 | 0.9 |  | 132 | 0.2 |  | 357 | 0.5 |
|  | Disease in child currently under treatment | No | 57839 | 73.3 |  | 8105 | 10.3 |  | 1707 | 2.2 |
|  |  | Yes | 6840 | 8.7 |  | 1106 | 1.4 |  | 326 | 0.4 |
|  |  | NA | 2180 | 2.8 |  | 335 | 0.4 |  | 459 | 0.6 |
|  | How the mother became pregnant this time | Spontaneously | 62042 | 78.6 |  | 8891 | 11.3 |  | 2323 | 2.9 |
|  |  | By ovulation induction through medication | 1784 | 2.3 |  | 243 | 0.3 |  | 61 | 0.1 |
|  |  | By AIH | 626 | 0.8 |  | 97 | 0.1 |  | 25 | 0.0 |
|  |  | By IVF | 1116 | 1.4 |  | 152 | 0.2 |  | 28 | 0.0 |
|  |  | By ICSI | 518 | 0.7 |  | 71 | 0.1 |  | 19 | 0.0 |
|  |  | By fresh embryo transfer | 32 | 0.0 |  | 1 | 0.0 |  | 0 | 0.0 |
|  |  | By frozen embryo transfer | 374 | 0.5 |  | 44 | 0.1 |  | 12 | 0.0 |
|  |  | By blastocyst transfer | 61 | 0.1 |  | 13 | 0.0 |  | 2 | 0.0 |
|  |  | NA | 306 | 0.4 |  | 34 | 0.0 |  | 22 | 0.0 |
|  | Recurrent miscarriage | No | 64021 | 81.1 |  | 9123 | 11.6 |  | 2343 | 3.0 |
|  |  | Yes | 666 | 0.8 |  | 87 | 0.1 |  | 24 | 0.0 |
|  |  | NA | 2172 | 2.8 |  | 336 | 0.4 |  | 125 | 0.2 |
|  | Emotional abuse from partner during pregnancy | No | 58661 | 74.4 |  | 7109 | 9.0 |  | 1779 | 2.3 |
|  |  | Yes | 7527 | 9.5 |  | 2301 | 2.9 |  | 372 | 0.5 |
|  |  | NA | 671 | 0.9 |  | 136 | 0.2 |  | 341 | 0.4 |
|  | Physical abuse from partner during pregnancy | No | 65706 | 83.3 |  | 9172 | 11.6 |  | 2111 | 2.7 |
|  |  | Yes | 561 | 0.7 |  | 264 | 0.3 |  | 51 | 0.1 |
|  |  | NA | 592 | 0.8 |  | 110 | 0.1 |  | 330 | 0.4 |
|  | Gestational Weight Gain | Normal weight gain | 34413 | 43.6 |  | 4734 | 6.0 |  | 1125 | 1.4 |
|  |  | Abnormal weight gain | 30923 | 39.2 |  | 4617 | 5.9 |  | 1177 | 1.5 |
|  |  | NA | 1523 | 1.9 |  | 195 | 0.2 |  | 190 | 0.2 |
|  | Psycological Distress during pregnancy | K6 <13 | 65108 | 82.5 |  | 8403 | 10.7 |  | 2291 | 2.9 |
|  |  | K6 >= 13 | 1044 | 1.3 |  | 989 | 1.3 |  | 102 | 0.1 |
|  |  | NA | 707 | 0.9 |  | 154 | 0.2 |  | 99 | 0.1 |
| *Weight-loss methods before pregnancy* | |  |  |  |  |  |  |  |  |  |
|  | Eating two-thirds as much as usual or less | No | 58174 | 73.7 |  | 7750 | 9.8 |  | 1879 | 2.4 |
|  |  | Yes | 8338 | 10.6 |  | 1739 | 2.2 |  | 315 | 0.4 |
|  |  | NA | 347 | 0.4 |  | 57 | 0.1 |  | 298 | 0.4 |
|  | Avoiding snacking and having a midnight snack | No | 41648 | 52.8 |  | 5617 | 7.1 |  | 1399 | 1.8 |
|  |  | Yes | 24864 | 31.5 |  | 3872 | 4.9 |  | 795 | 1.0 |
|  |  | NA | 347 | 0.4 |  | 57 | 0.1 |  | 298 | 0.4 |
|  | Eating specific diet food | No | 60295 | 76.4 |  | 8252 | 10.5 |  | 1990 | 2.5 |
|  |  | Yes | 6217 | 7.9 |  | 1237 | 1.6 |  | 204 | 0.3 |
|  |  | NA | 347 | 0.4 |  | 57 | 0.1 |  | 298 | 0.4 |
|  | Taking a diet pill | No | 65314 | 82.8 |  | 9159 | 11.6 |  | 2148 | 2.7 |
|  |  | Yes | 1198 | 1.5 |  | 330 | 0.4 |  | 46 | 0.1 |
|  |  | NA | 347 | 0.4 |  | 57 | 0.1 |  | 298 | 0.4 |
|  | Vomiting after eating | No | 65702 | 83.3 |  | 9204 | 11.7 |  | 2154 | 2.7 |
|  |  | Yes | 810 | 1.0 |  | 285 | 0.4 |  | 40 | 0.1 |
|  |  | NA | 347 | 0.4 |  | 57 | 0.1 |  | 298 | 0.4 |
|  | Smoking cigarettes | No | 64011 | 81.1 |  | 8731 | 11.1 |  | 2044 | 2.6 |
|  |  | Yes | 2501 | 3.2 |  | 758 | 1.0 |  | 150 | 0.2 |
|  |  | NA | 347 | 0.4 |  | 57 | 0.1 |  | 298 | 0.4 |
|  | Exercise | No | 45666 | 57.9 |  | 6041 | 7.7 |  | 1523 | 1.9 |
|  |  | Yes | 20846 | 26.4 |  | 3448 | 4.4 |  | 671 | 0.9 |
|  |  | NA | 347 | 0.4 |  | 57 | 0.1 |  | 298 | 0.4 |

NA, Not Answered; EPDS, Edinburgh Postnatal Depression Scale; PPD, postpartun depression; BMI, Body Mass Index; AIH, Artificial Insemination of Husband; IVF, In Vitro Fertilization; ICSI, Intracytoplasmic Sperm Injection; K6, T he Kessler 6-Item Psychological Distress Scale.

**Supplemental Table 2.** Odds ratio (95% CI) of PPD to use of weight-loss methods after stratified by BMI.

| BMI stratified | Group | *N* | aORa | Lower 95% CI | Higher 95% CI | p-value |  |
| --- | --- | --- | --- | --- | --- | --- | --- |
| underweight women | reference | 9010 | 1.000 |  |  |  |  |
|  | Method1 | 716 | 1.478 | 1.190 | 1.826 | 0.000 | X5 |
|  | reference | 7416 | 1.000 |  |  |  |  |
|  | Method2 | 2310 | 1.235 | 1.067 | 1.427 | 0.004 |  |
|  | reference | 9399 | 1.000 |  |  |  |  |
|  | Method3 | 327 | 1.682 | 1.246 | 2.244 | 0.001 |  |
|  | reference | 9631 | 1.000 |  |  |  |  |
|  | Method4 | 95 | 1.066 | 0.603 | 1.810 | 0.819 |  |
|  | reference | 9607 | 1.000 |  |  |  |  |
|  | Method5 | 119 | 1.521 | 0.938 | 2.399 | 0.079 |  |
|  | reference | 9389 | 1.000 |  |  |  |  |
|  | Method6 | 337 | 1.553 | 1.159 | 2.061 | 0.003 |  |
|  | reference | 7967 | 1.000 |  |  |  |  |
|  | Method7 | 1759 | 1.145 | 0.974 | 1.343 | 0.098 |  |
|  |  |  |  |  |  |  |  |
| normal-weight women | reference | 39774 | 1.000 |  |  |  |  |
|  | Method1 | 6171 | 1.340 | 1.237 | 1.449 | 0.000 |  |
|  | reference | 27610 | 1.000 |  |  |  |  |
|  | Method2 | 18335 | 1.074 | 1.011 | 1.141 | 0.020 |  |
|  | reference | 41451 | 1.000 |  |  |  |  |
|  | Method3 | 4494 | 1.233 | 1.126 | 1.349 | 0.000 |  |
|  | reference | 45046 | 1.000 |  |  |  |  |
|  | Method4 | 899 | 1.358 | 1.135 | 1.617 | 0.001 |  |
|  | reference | 45303 | 1.000 |  |  |  |  |
|  | Method5 | 642 | 1.847 | 1.514 | 2.242 | 0.000 |  |
|  | reference | 44083 | 1.000 |  |  |  |  |
|  | Method6 | 1862 | 1.395 | 1.229 | 1.580 | 0.000 |  |
|  | reference | 30518 | 1.000 |  |  |  |  |
|  | Method7 | 15427 | 1.132 | 1.064 | 1.204 | 0.000 |  |
|  |  |  |  |  |  |  |  |
| obese women | reference | 5181 | 1.000 |  |  |  |  |
|  | Method1 | 1208 | 1.176 | 0.976 | 1.411 | 0.084 | X5 |
|  | reference | 3456 | 1.000 |  |  |  |  |
|  | Method2 | 2933 | 1.149 | 0.987 | 1.339 | 0.074 |  |
|  | reference | 5098 | 1.000 |  |  |  |  |
|  | Method3 | 1291 | 1.186 | 0.988 | 1.419 | 0.065 |  |
|  | reference | 6158 | 1.000 |  |  |  |  |
|  | Method4 | 231 | 1.220 | 0.845 | 1.729 | 0.275 |  |
|  | reference | 6299 | 1.000 |  |  |  |  |
|  | Method5 | 90 | 1.379 | 0.778 | 2.343 | 0.251 |  |
|  | reference | 6030 | 1.000 |  |  |  |  |
|  | Method6 | 359 | 1.507 | 1.132 | 1.989 | 0.004 |  |
|  | reference | 3828 | 1.000 |  |  |  |  |
|  | Method7 | 2561 | 1.137 | 0.973 | 1.327 | 0.105 |  |
| Cl, confidence interval; aOR, adjusted odds ratio; PPD, postpartum depression; BMI, body mass index. | | | | | | | |
| ^a^Adjusted for age of the mother, sex, mode of delivery, number of previous deliveries, marital status, feelings when learning about pregnancy, stressful events, emotional support, highest level of education, annual household income, emotional abuse from partner, physical abuse from partner, BMI in first trimester, disease in the child currently under treatment, how the mother became pregnant this time, recurrent miscarriage, and gestational weight gain. | | | | | | | |

**Supplemental Table 3.** Odds ratio (95% CI) of PPD to weight-loss method score after stratified by BMI.

| BMI stratified | Group | *N* | aOR^a^ | Lower 95% CI | Higher 95% CI | p-value |
| --- | --- | --- | --- | --- | --- | --- |
| underweight women | reference (score = 0) | 6173 | 1.000 |  |  |  |
|  | 1 | 1822 | 1.310 | 1.106 | 1.547 | 0.002 |
|  | 2 | 725 | 1.260 | 0.989 | 1.593 | 0.057 |
|  | 3 | 404 | 1.849 | 1.402 | 2.417 | 0.000 |
|  | 4 | 278 | 1.421 | 0.993 | 1.994 | 0.048 |
|  | 5 | 98 | 1.561 | 0.875 | 2.650 | 0.113 |
|  | 6 | 89 | 2.084 | 1.189 | 3.514 | 0.008 |
|  | 7 | 137 | 1.844 | 1.161 | 2.855 | 0.008 |
|  |  |  |  |  |  |  |
| normal-weight women | reference (score = 0) | 18367 | 1.000 |  |  |  |
|  | 1 | 12039 | 1.197 | 1.108 | 1.293 | 0.000 |
|  | 2 | 6548 | 1.180 | 1.075 | 1.295 | 0.001 |
|  | 3 | 3161 | 1.344 | 1.196 | 1.508 | 0.000 |
|  | 4 | 2907 | 1.415 | 1.256 | 1.592 | 0.000 |
|  | 5 | 895 | 1.862 | 1.552 | 2.224 | 0.000 |
|  | 6 | 1071 | 1.495 | 1.253 | 1.774 | 0.000 |
|  | 7 | 957 | 1.679 | 1.407 | 1.995 | 0.000 |
|  |  |  |  |  |  |  |
| obese women | reference (score = 0) | 1766 | 1.000 |  |  |  |
|  | 1 | 1595 | 1.340 | 1.071 | 1.679 | 0.011 |
|  | 2 | 1078 | 1.322 | 1.031 | 1.692 | 0.027 |
|  | 3 | 646 | 1.350 | 1.014 | 1.787 | 0.038 |
|  | 4 | 609 | 1.577 | 1.187 | 2.086 | 0.002 |
|  | 5 | 235 | 1.529 | 1.027 | 2.238 | 0.032 |
|  | 6 | 253 | 2.104 | 1.465 | 2.987 | 0.000 |
|  | 7 | 207 | 1.519 | 1.001 | 2.265 | 0.044 |
| Cl, confidence interval; aOR, adjusted odds ratio; PPD, postpartum depression; BMI, body mass index. | | | | | | |
| ^a^Adjusted for age of the mother, sex, mode of delivery, number of previous deliveries, marital status, feelings when learning about pregnancy, stressful events, emotional support, highest level of education, annual household income, emotional abuse from partner, physical abuse from partner, BMI in first trimester, disease in the child currently under treatment, how the mother became pregnant this time, recurrent miscarriage, and gestational weight gain. | | | | | | |

**Supplemental Table 4.** Odds ratio (95% CI) of PPD according to use of weight-loss methods after removing participants with a medical history of psychiatric disorders using missing value complemented dataset.

|  |  | aOR | std.error | 2.50% | 97.50% | p.value |
| --- | --- | --- | --- | --- | --- | --- |
| K6 score < 13 | Using at least one weight-loss method | 1.31 | 0.02 | 1.25 | 1.38 | <0.001 |
| K6 score ≥13 | Using at least one weight-loss method | 1.19 | 0.10 | 0.98 | 1.45 | 0.07 |

Cl, confidence interval; OR, odds ratio; PPD, postpartun depression.

a:Adjusted for age of mother, sex, mode of delivery, number of previous deliveries, marital status, feelings when learning about pregnancy, stressful events, emotional support, highest level of education, annual household income, emotional abuse from partner, physical abuse from partner, BMI before pregnancy, disease in child currently under treatment, how the mother became pregnant this time, and recurrent miscarriage.

**Supplemental Table 5.** Odds ratio (95% CI) of PPD according to each weight-loss method using missing value complemented dataset.

|  | aOR | std.error | 2.50% | 97.50% | p.value |
| --- | --- | --- | --- | --- | --- |
| Eating two-thirds as much as usual or less | 1.32 | 0.03 | 1.24 | 1.40 | <0.001 |
| Avoiding eating between meals and having a midnight snack | 1.10 | 0.02 | 1.05 | 1.16 | <0.001 |
| Eating specific diet food | 1.22 | 0.04 | 1.14 | 1.31 | <0.001 |
| Taking a diet pill | 1.33 | 0.07 | 1.17 | 1.52 | <0.001 |
| Vomiting after eating | 1.78 | 0.08 | 1.54 | 2.06 | <0.001 |
| Smoking cigarettes | 1.46 | 0.05 | 1.33 | 1.60 | <0.001 |
| Exercise | 1.16 | 0.02 | 1.10 | 1.21 | <0.001 |

a: Adjusted for age of mother, sex, mode of delivery, number of previous deliveries, marital status, feelings when learning about pregnancy, stressful events, emotional support, highest level of education, annual household income, emotional abuse from partner, physical abuse from partner, BMI before pregnancy, disease in child currently under treatment, how the mother became pregnant this time, and recurrent miscarriage.

**Supplementary Figure Legends**

**Supplementary Fig. 1** Association between weight-loss methods before pregnancy and PPD in BMI-stratified analysis using missing value complemented dataset in **(a):** underweight women, **(b):** normal weight women and **(c):** obese women. Error bar represents 95% confidence interval. Dotted line indicates the reference category (women who did not perform any weight-loss behaviors). Adjusted for age of the mother, child’s sex, mode of delivery, number of previous deliveries, marital status, feelings when learning about the pregnancy, stressful events, emotional support, highest level of education, annual household income, emotional abuse from partner, physical abuse from partner, diseases in the child currently under treatment, how the mother became pregnant this time, recurrent miscarriage, psychological distress and gestational weight gain. PPD, postpartum depression; BMI, body mass index.

**Supplementary Fig. 2** Risk of postpartum depression was increased with weight-loss method score in a dependent manner. **(a):** Association between weight-loss method score before pregnancy and PPD in underweight women. **(b):** Association between weight-loss method score before pregnancy and PPD in normal weight women **(c):** Association between weight-loss method score before pregnancy and PPD in obese women. Error bar represents 95% confidence interval. Dotted line indicates the reference category (women who did not perform any weight-loss behaviors). Adjusted for age of the mother, child’s sex, mode of delivery, number of previous deliveries, marital status, feelings when learning about the pregnancy, stressful events, emotional support, highest level of education, annual household income, emotional abuse from partner, physical abuse from partner, diseases in the child currently under treatment, how the mother became pregnant this time, recurrent miscarriage, psychological distress and gestational weight gain.

**Supplementary Fig. 1**


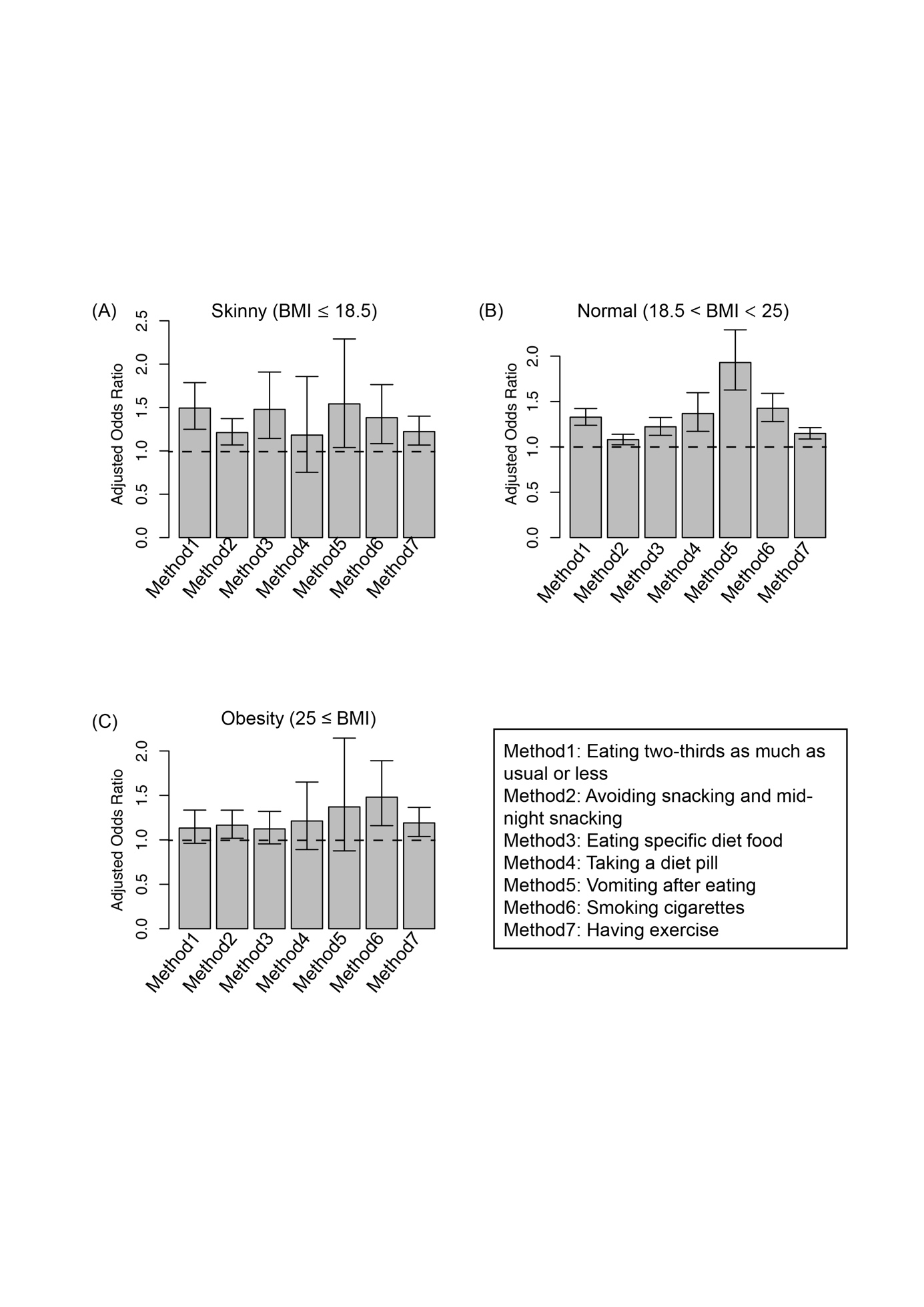


**Supplementary Fig. 2**


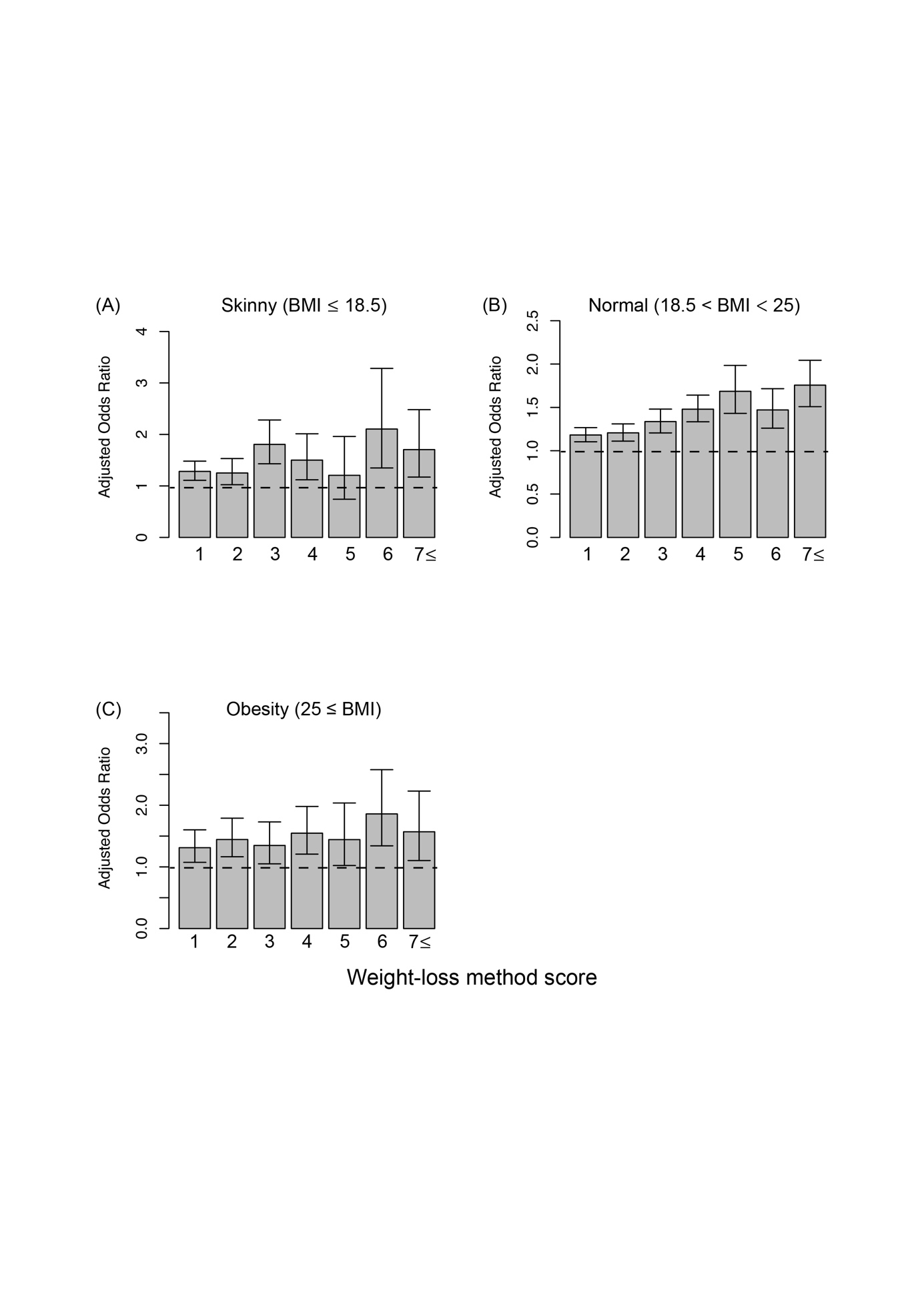

Supplement: Supplementary file 1 — Supplementary Information. [file 41598_2023_34547_MOESM1_ESM.docx]
